# Identification of a gene signature associated with radiotherapy and prognosis in gliomas

**DOI:** 10.18632/oncotarget.21634

**Published:** 2017-10-06

**Authors:** Shu Li, Juanhong Shi, Hongliang Gao, Yan Yuan, Qi Chen, Zhenyu Zhao, Xiaoqiang Wang, Bin Li, LinZhao Ming, Jun Zhong, Ping Zhou, Hua He, Bangbao Tao, Shiting Li

**Affiliations:** ^1^ Department of Pathophysiology, Wannan Medical College, Wuhu 241002, China; ^2^ Department of Neurosurgery, Xinhua Hospital, School of Medicine, Shanghai Jiaotong University, Shanghai 200092, China; ^3^ Department of Pathology Neurosurgery, Xinhua Hospital, School of Medicine, Shanghai Jiaotong University, Shanghai 200092, China; ^4^ Department of Anesthesiology, Xinhua Hospital, School of Medicine, Shanghai Jiaotong University, Shanghai 200092, China; ^5^ Department of Neurosurgery, Changzheng Hospital, The Second Hospital Affiliated with The Second Military Medical University, Shanghai 200092, China

**Keywords:** glioma, prognosis, gene signature

## Abstract

Glioma is one of the most common primary brain tumors with poor prognosis. Although radiotherapy is an important treatment method for gliomas, the efficacy is still limited by the high occurrence of radioresistance and the underlying molecular mechanism is unclear. Here, we performed a data mining work based on four glioma expression datasets. These datasets were classified into training set and validation set. Radiotherapy-induced differential expressed genes and prognosis-associated genes were screened using different classifiers. The Kaplan-Meier curves along with the two-sided Log Rank (Mantel-Cox) test were used to evaluate overall survival. We found the gene expression profiles of gliomas between those patients received radiotherapy and those patients without received radiotherapy were quite different. A 20-gene signature was identified, which was associated with radiotherapy.Furthermore, a novel 5-gene signature (*HOXC10*, *LOC101928747*, *CYB561D2*, *RPL36A* and *RPS4XP2*) as an independent predictor of glioma patients’ prognosis was further derived from the 20-gene signature. These findings provided a new insight into the molecular mechanism of radioresistance in gliomas. The 5-gene signature might represent therapeutic target for gliomas.

## INTRODUCTION

Glioma is one of the most common primary brain tumors in adults and malignant gliomas, accounting for approximately 70% of malignant primary brain tumors [1, 2]. According to the World Health Organization (WHO) classification based on four main features: nuclear atypia, mitoses, microvascular proliferation, and necrosis, gliomas are classified as: grade I (pilocytic astrocytomas, PA), grade II (low grade), grade III (anaplastic) and grade IV (glioblastoma, GBM) [3]. Recently, there have been important advances in understanding the molecular pathogenesis of malignant gliomas [4] and significant progress in its treatment [5]. However, the overall survival of gliomas remains poor. The median survival time is only 12 to 15 months for patients with GBM and 2 to 5 years for patients with anaplastic gliomas [1]. Radiotherapy with ionizing radiation (IR) is used for the treatment of low grade gliomas [6] and GBM [7]. However, its efficacy is often limited by the occurrence of radioresistance [8] and the heterogeneity of gliomas with different histological subtypes and grades [9]. Furthermore, the molecular mechanism responsible for the radioresistance of human gliomas is still unclear. Exploration of the molecular alterations after radiotherapy may provide comprehensive understanding of radioresistance in gliomas. In this study, we attempt to find a gene signature associated with radiotherapy and prognosis in gliomas. After downloading microarray data sets of gliomas from the Gene Expression Omnibus (GEO) database and TCGA database, and analyzing the differentially expressed genes (DEGs) with different classifiers between glioma samples that received radiotherapy and that did not receive radiotherapy, we successfully obtained a 20-gene signature that was associated to radiotherapy. Then we further identified a 5-gene signature from the 20-gene signature which was predictive for the prognosis of glioma patients in different data sets.

## RESULTS

### A 20-gene signature associated with radiotherapy in gliomas

**Figure 1:**
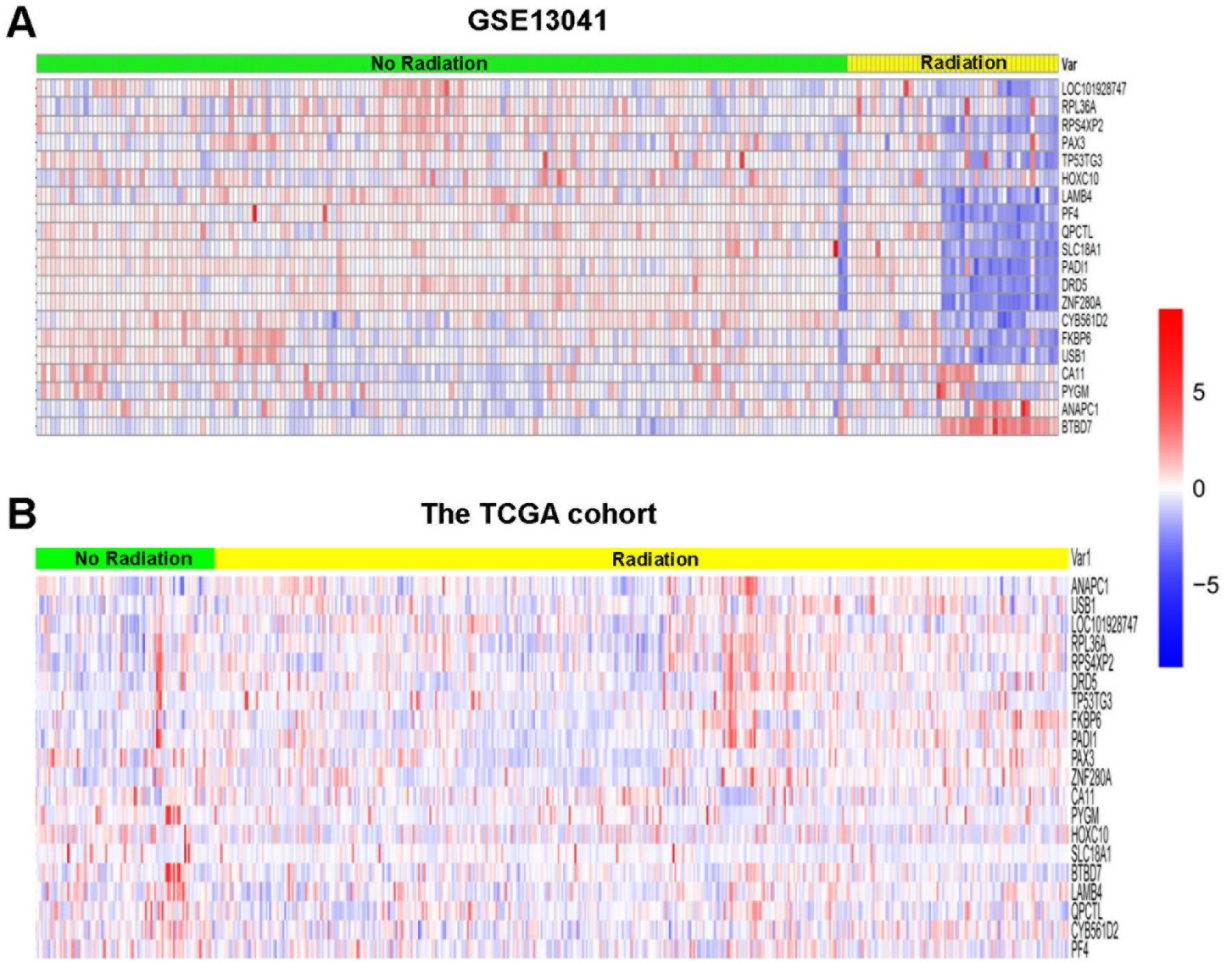
Hierarchical clustering analysis of GSE13041 and the TCGA cohort **(A)** Result of GSE13041. **(B)** Result of the TCGA cohort. Rows represent genes, and columns represent patients. Red, high expression; blue, low expression, according to Z scores.

**Figure 2:**
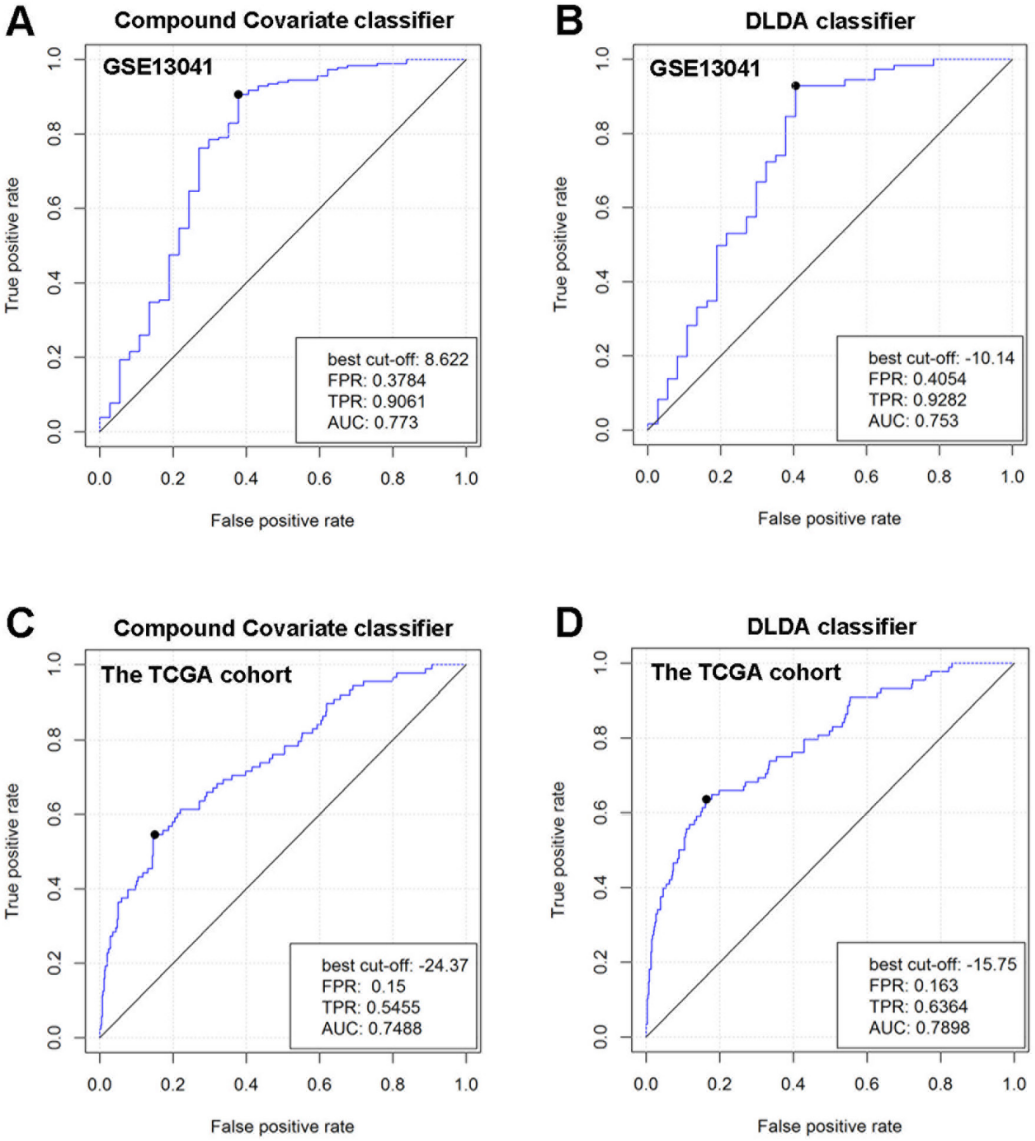
Comprehensive ability of the 20-gene signature to separate radiation group and no radiation group in GSE13041 and the TCGA cohort The ROC curves were used in 2 different linear classifiers (DLDA classifier and Compound Covariate classifier). **(A)** ROC curve for Compound Covariate classifier in GSE13041. **(B)** ROC curve for DLDA classifier in GSE13041. **(C)** ROC curve for Compound Covariate classifier in the TCGA cohort. **(D)** ROC curve for DLDA classifier in the TCGA cohort.

To explore gene markers associated with radiotherapy in gliomas, data mining was conducted. Three data sets were divided into a training set (GSE13041) including 218 patients and 2 validation sets (GSE7696 and TCGA cohort) including 628 patients. First, we used 5 different classifiers to re-classify the clinical samples into radiation group and no radiation group in the training set. As a result, we identified a 20-gene signature (*ANAPC1*, *BTBD7, CA11, CYB561D2, DRD5, FKBP6, HOXC10, LAMB4, LOC101928747, PADI1, PAX3, PF4, PYGM, QPCTL, RPL36A, RPS4XP2, SLC18A1, TP53TG3, USB1, ZNF280A* in Supplementary Table 1) that was associated with radiotherapy in gliomas. In detail, the 20-gene signature could re-classify the two groups with high accuracy between 78% and 87%, high specificity between 0.796 and 0.928, and high negative predictive value (NPV) between 0.851 and 0.917 in different classifiers (Table 1), indicating relative high efficiency of this gene signature to distinguish glioma patients receiving radiotherapy from patients not receiving radiotherapy. When the hierarchical clustering analysis was conducted, we also found different expression pattern of the 20 genes between radiation group and no radiation group (Figure 1A). Furthermore, we used the receiver operating characteristic (ROC) curves to evaluate the comprehensive ability of this gene signature in the 2 linear classifiers (Compound Covariate classifier and DLDA classifier) to separate these two groups. As a result, the 20-gene signature could separate these two groups with AUC value of 0.773 in Compound Covariate classifier (Figure 2A) and 0.753 in DLDA classifier (Figure 2B), respectively. This result further indicated moderate ability of this gene signature to separate these two groups in the training set. Afterwards, we used the validation sets to verify the result derived from the training set. We also found high accuracy between 66% and 88% in different classifiers in the TCGA cohort, and high accuracy between 66% and 99% in different classifiers in the GSE7696, respectively (Table 2). In the hierarchical clustering analysis, we found similar differential expression pattern of the 20 genes between radiation group and no radiation group in the TCGA cohort as that in the training set (Figure 1B). Also, moderate ability of the 20-gene signature to separate these two groups was detected in the TCGA cohort with AUC value of 0.749 in Compound Covariate classifier (Figure 2C) and 0.790 in DLDA classifier (Figure 2D), respectively.

**Table 1:** The ability of the 20-gene signature in separating the radiation group from the no radiation group in different classifiers in the training set GSE13041

Classifier	Sensitivity	Specificity	PPV	NPV	Accuracy(%)
Compound covariate	0.541	0.928	0.606	0.908	86
DLDA	0.459	0.928	0.567	0.894	85
1-Nearest Neighbor	0.243	0.884	0.300	0.851	78
3-Nearest Neighbor	0.270	0.917	0.400	0.860	81
Nearest Centroid	0.622	0.851	0.460	0.917	81
Bayesian CCP	0.432	0.796	0.302	0.873	87

DLDA: Diagonal Linear Discriminant Analysis; PPV: positive predictive value; NPV: negative predictive value.

**Table 2:** The accuracy of the 20-gene signature in separating the radiation group from the no radiation group in different classifiers in the validation sets (GSE7696 and the TCGA cohort)

Classifier	The TCGA cohort	GSE7696
	Accuracy(%)	Accuracy(%)
Compound covariate	84	66
DLDA	66	99
1-Nearest Neighbor	86	86
3-Nearest Neighbor	88	88
Nearest Centroid	88	80
Bayesian CCP	86	95

### Identification of a 5-gene signature related to prognosis of glioma patients

**Figure 3:**
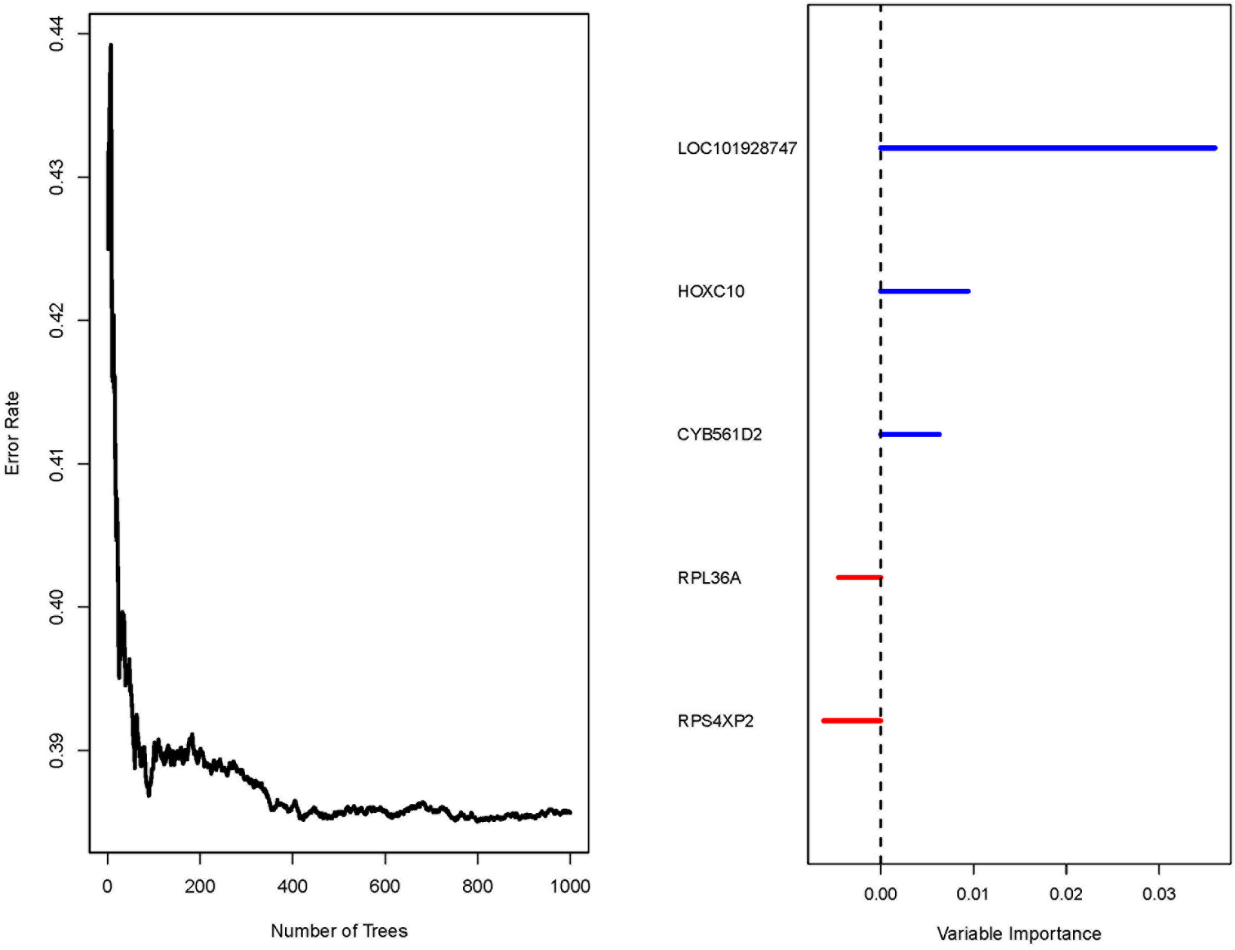
Result of the random survival forests algorithm in GSE13041 Left: Error rate of the function tree; Right: variable importance values for each of the 5 gene.

**Figure 4:**
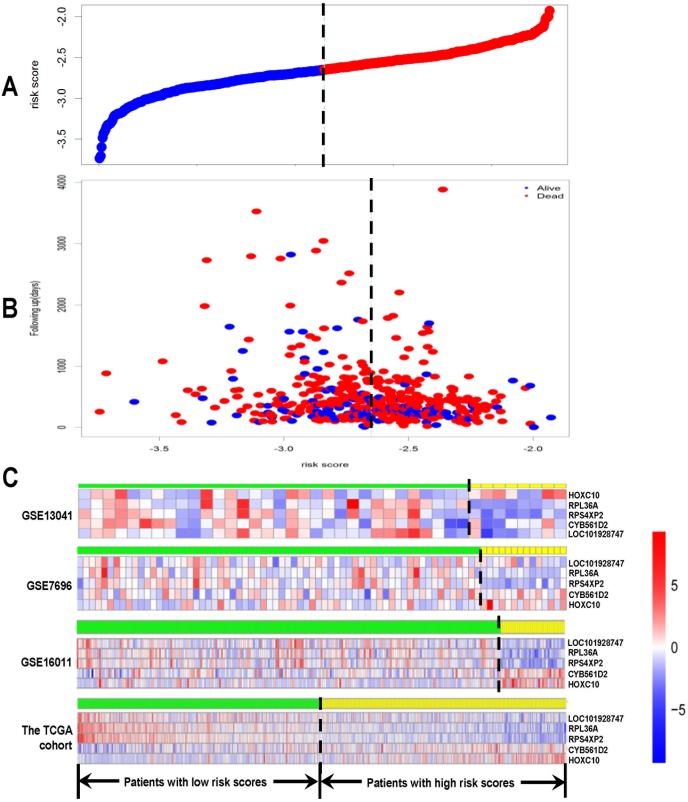
Risk score analysis of GSE13041, GSE7696, GSE16011 and the TCGA cohort The distribution of risk score based on the 5-gene signature, patients’ survival status and the 5-gene expression profiles were analyzed in each of the 4 data sets. **(A)** Risk score distribution of all the patients in the 4 data sets; **(B)** patients’ survival status and time of all the patients in the 4 data sets; **(C)** heatmap of the 5-gene expression profiles. Rows represent genes, and columns represent patients. Red, high expression; blue, low expression, according to Z scores. The black dotted line represents the median risk score. According to the median risk score, patients were divided into lowrisk and high-risk groups.

**Figure 5:**
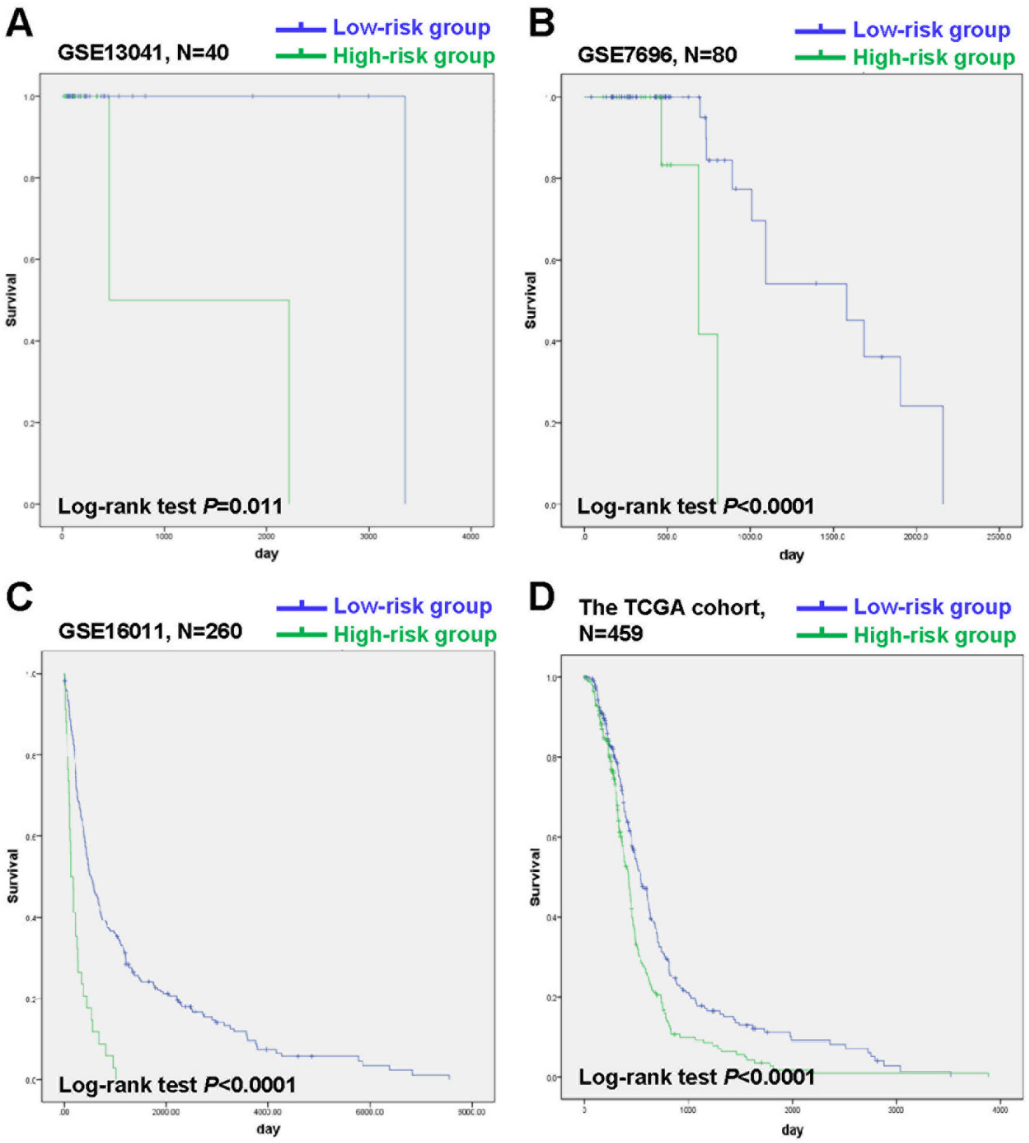
Kaplan-Meier analysis of OS of patients in the low-risk group and the high-risk group in GSE13041, GSE7696, GSE16011 and the TCGA cohort **(A)** Kaplan-Meier curves in GSE13041 (high risk=8, low risk=32). **(B)** Kaplan-Meier curves in GSE7696 (high risk=14, low risk=66). **(C)** Kaplan-Meier curves in GSE16011 (high risk=34, low risk=226). **(D)** Kaplan-Meier curves in the TCGA cohort (high risk=230, low risk=229). The tick marks on the Kaplan-Meier curves represent the censored subjects.

A 20-gene signature associated with radiotherapy in gliomas has been identified, suggesting that the expression changes of these genes in gliomas might be induced by radiotherapy. We further hypothesize that some of them might be associated with radioresistance in gliomas and thus could influence the prognosis of the patients. Next, we tried to screen genes which were related to the prognosis of glioma patients from the 20-gene signature. In order to achieve this goal, GSE13041 was also used as the training set while GSE7696, GSE16011 and the TCGA cohort were used as the validation sets. First, when univariable Cox proportional hazards regression analysis was used in the training set, we obtained 5 genes (*HOXC10, LOC101928747, CYB561D2, RPL36A and RPS4XP2*) which were highly associated with patients’ prognosis from the 20-gene signature (Table 3). The random survival forests algorithm further validated that all the 5 genes were important for survival of glioma patients when the cut value of relative importance was set as 0.1 (Figure 3 and Table 3). Then we successfully constructed a risk score model according to the expression levels of these 5 genes as follows: Risk score = 0.469×*CYB561D2* + 0.197×*HOXC10* - 0.066×*RPS4XP2* - 0.506×*RPL36A* - 0.645×*LOC101928747*. Next, all patients in the training set and the validation sets were divided into the high-risk group and the low-risk group according to the median risk score. The distribution of risk scores and the survival status of all the patients in the 4 data sets were showed in Figure 4A, 4B. We found that the radio of alive patients over dead patients at the endpoint of follow-up in the low-risk group was significantly higher than that in the high-risk group (Likelihood ratio test, p=9.138e-05). When Kaplan-Meier curves were used to further evaluate the difference of overall survival (OS) between the two groups, we found patients in the high-risk group had significantly shorter OS than those in the low-risk group (Log Rank test, P=0.011 in GSE13041, P<0.0001 in GSE7696, GSE16011, and the TCGA cohort) (Figure 5A, 5B). These results indicated that the 5-gene signature was indeed associated with the prognosis of glioma patients.

**Table 3:** A 5-gene signature identified from the 20-gene by univariable Cox proportional hazards regression analysis and the random survival forests algorithm

Gene	Univariable Cox proportional hazards regression analysis			The random survival forests algorithm	
	Parametric *P*-value	FDR	Hazard ratio	Variable importance	Relative importance
HOXC10	< 1×10^-7^	< 1×10^-7^	1.521	0.0094	0.2614
LOC101928747	< 1×10^-7^	1×10^-6^	0.423	0.036	1
CYB561D2	6×10^-7^	4×10^-6^	2.247	0.0063	0.175
RPL36A	9×10^-7^	4.5×10^-6^	0.542	-0.0045	-0.1253
RPS4XP2	2×10^-6^	8×10^-6^	0.605	-0.0061	-0.1701

### Prognosis prediction by the 5-gene signature is independent of clinical and pathological factors

To assess whether the prognosis prediction ability of the 5-gene signature is independent of other clinical or pathological factors of the patients with gliomas, univariate and multivariable Cox regression analysis was performed in GSE13041, GSE7696, GSE16011 and the TCGA cohort. As shown in Table 4, univariable and multivariable Cox regression analysis both indicated that the risk score was significantly associated with poor prognosis of glioma patients in most data sets (GSE7696, GSE16011 and the TCGA cohort) (for GSE7696, HR=13.20, 95% CI 2.58 to 67.57, P=0.0020 in univariable model, and HR=21.61, 95% CI 2.99 to 156.07, P=0.0020 in multivariable model; for GSE16011, HR=3.27, 95% CI 2.24 to 4.97, P=9.80×10-10 in univariable model, and HR=2.16, 95% CI 1.19 to 3.90, P=0.0010 in multivariable model; for the TCGA cohort, HR=2.23, 95% CI 1.51 to 3.30, P=9.33E-05 in univariable model, and HR=1.69, 95% CI 1.08 to 2.63, P=0.022 in multivariable model), though not significantly in GSE13041 (HR=0.946, 95% CI 0.392 to 2.281, P=0.901 in univariable model, and HR=0.75, 95% CI 0.28 to 2.04, P=0.58 in multivariable model). These results indicated that the risk score based on the 5-gene signature might be an independent predictor of glioma patients’ survival.

**Table 4:** Results of univariate and multivariable Cox regression analysis of GSE13041

Parameters	Univariable model				Multivariable model	
	HR	95%CI of HR	*P* value	HR	95%CI of HR	*P* value
**GSE13041**						
Risk_score	546.45	0-38801829122.39	0.49	0.75	0.28-2.04	0.58
Age	1.01	0.99-1.03	0.36	1.03	1.00-1.05	0.056
HC	0.96	0.66-1.39	0.83	0.91	0.64-1.30	0.61
HC_coded	0.48	0.23-0.99	5.00×10^-2^	0.36	0.16-0.81	0.013
Gender	0.68	0.33-1.38	0.28	0.49	0.22-1.06	0.069
Chemotx_administered_prior_to_tumor_resection	1.08	0.47-2.50	0.85	1.56	0.51-4.75	0.43
Temodar_administered_prior_to_tumor_resection	1.22	0.62-2.40	0.56	1.47	0.58-3.70	0.42
FUFA	2.90	0.39-21.35	0.30	8.09	0.96-68.00	0.054
**GSE7696**						
Risk_score	13.20	2.58-67.57	0.0020	21.61	2.99-156.07	0.0020
Disease_status	0.15	0.019-1.20	0.074	0.13	0.011-1.48	0.10
Age	1.04	0.96-1.13	0.32	1.09	0.99-1.21	0.095
Gender	0.35	0.095-1.27	0.11	0.41	0.077-2.18	0.30
Mgmt	2.12	0.22-20.73	0.52	12.44	0.65-238.48	0.094
**GSE16011**						
Risk_score	3.27	2.24-4.79	9.80×10^-10^	2.16	1.19-3.90	0.0010
Gender	1.08	0.82-1.42	0.60	0.82	0.55-1.22	0.33
Histological diagnosis	0.86	0.81-0.92	3.44×10^-6^	0.84	0.76-0.94	0.011
Age	1.04	1.03-1.05	1.01×10^-6^	1.03	1.01-1.04	0.046
KPS score	0.98	0.97-0.99	1.61×10^-7^	0.98	0.97-0.99	0.0030
Chemotherapy	0.81	0.57-1.13	0.21	0.94	0.53-1.65	0.82
IDH1_mutation	0.55	0.41-0.75	1.17×10^-4^	0.62	0.41-0.95	0.026
**The TCGA cohort**						
Risk_score	2.23	1.51-3.30	0	1.69	1.08-2.63	0.022
Gender	1.08	0.87-1.35	0.47	1.11	0.86-1.43	0.42
KPS score	0.99	0.98-0.99	0.0060	0.99	0.98-1.00	0.063
Age	1.02	1.01-1.03	5.24×10^-7^	1.02	1.01-1.03	0.0010

### Identification of the 5-gene signature associated biological pathways and processes by GSEA

**Figure 6:**
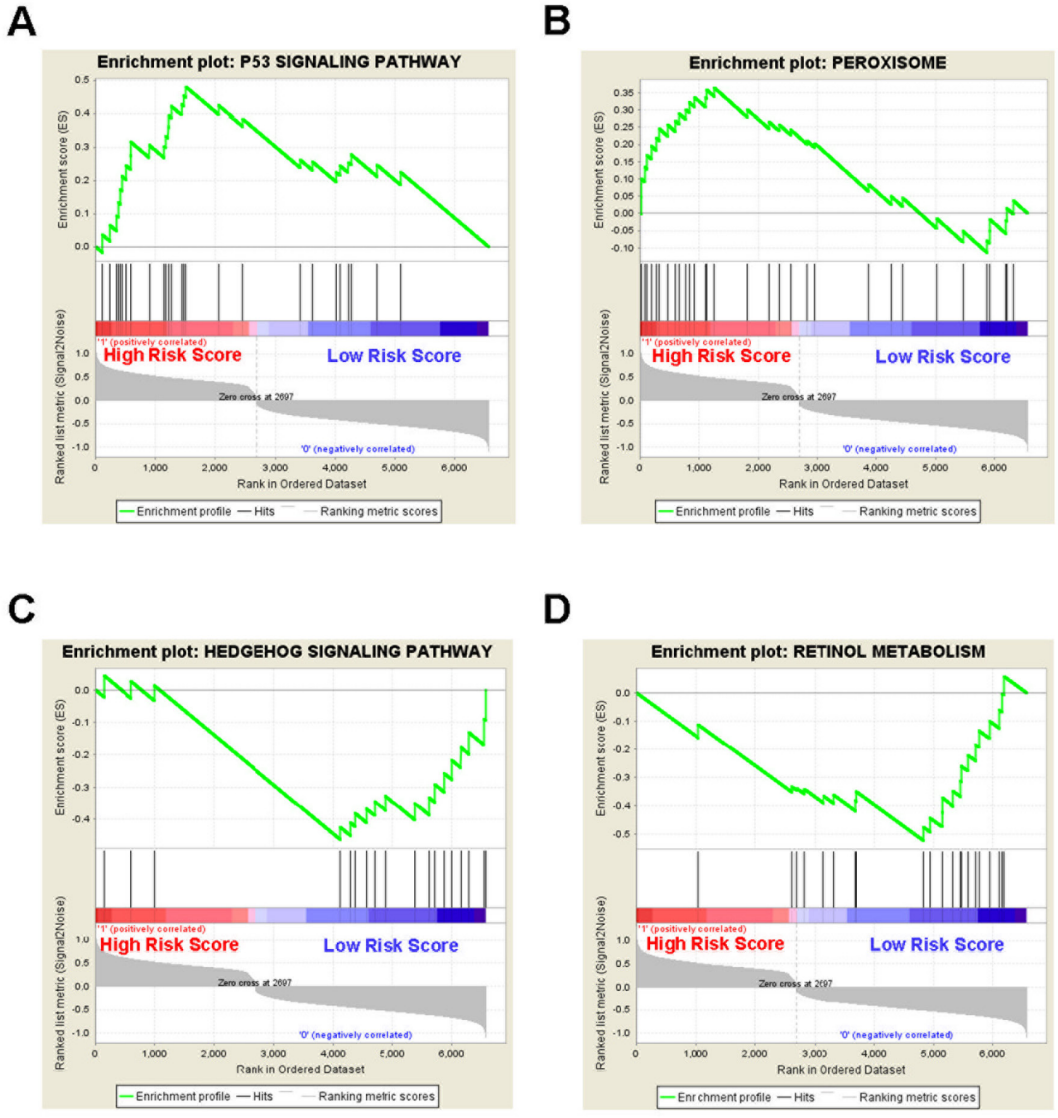
Gene set enrichment analysis reveals the 5-gene signature associated biological pathways and processes in the GSE13041 cohort GSEA validated **(A)** p53 signaling pathway and **(B)** peroxisome were enriched in the high-risk group, and **(C)** hedgehog signaling pathway and **(D)** retinol metabolism were enriched in the low-risk group.

To identify the 5-gene signature associated biological pathways and processes, Gene Set Enrichment Analysis (GSEA) was performed in the GSE13041 cohort. The gene expression profile in the high-risk group and low-risk group were compared. As a result, several cancer related pathways or processes such as p53 signaling pathway and peroxisome were enriched in the high-risk group, while cancer related pathways or processes such as hedgehog signaling pathway and retinol metabolism were enriched in the low-risk group (Figure 6).

## DISCUSSION

In this study, we examined the gene profiles of glioma tissues from patients receiving or not receiving radiotherapy and identified a 20-gene signature associated with radiotherapy in gliomas. Furthermore, a 5-gene signature associated with the prognosis of glioma patients was identified from the 20-gene signature. This 5-gene signature is also an independent predictor of glioma patients’ survival. Malignant tumors show massive molecular alterations including gene mutations and abnormal gene expression. The differentially expressed genes between tumors and normal tissues could be used as biomarkers of malignant tumors. However, single-gene biomarkers may result in low reproducibility across different data sets, while gene signature with a panel of genes may be superior to single-gene biomarkers [10]. In fact, the potential of gene signatures as biomarkers of malignant tumors have been widely explored since the pioneering study of molecular classification in acute myeloid leukemia (AML) [11]. In the following studies, gene signatures as classification markers [12, 13], diagnosis markers [14, 15], prognosis predictors [15–20] and markers of treatment response [21–23] were identified in different kinds of malignant tumors. As for gliomas, gene signatures were also identified for classification [24] and prognosis prediction [25]. However, few biomarkers are specific to radiotherapy or can indicate response to radiotherapy for glioma patients. In this study, we obtained a radiotherapy-specific 20-gene signature in gliomas, and further identified a 5-gene signature with prognostic value from the 20-gene signature, which may be used as new biomarkers for glioma patients receiving radiotherapy. As previously reported [10], the criteria to establish a gene signature as a marker of a particular treatment method or a prognosis predictor are as follows. First, the gene signature shows specific association with this treatment or patients’ prognosis. Second, the accuracy and reproducibility of the gene signature are demonstrated in independent data sets. Third, the gene signature is independent of other clinical factors in a multivariate analysis. Here, we first identified a 20-gene signature associated with radiotherapy in glioma patients. Then a 5-gene signature associated with patients’ survival was generated from the 20-gene signature. Both the association between the 20-gene signature and radiotherapy, and the prognostic value of the 5- gene signature were validated in training set and several validation sets. Moreover, the risk score based on the 5-gene signature was still significantly associated with poor prognosis of glioma patients in most data sets by in univariable and multivariable Cox regression analysis. Therefore, we identified promising and stringent gene signatures which could be used as reliable biomarkers in gliomas. Besides, single bioinformatics model is usually prone to false-positive candidate genes lacking real biological relevance or less clinical utility [26]. Also, the FDR (False Discovery Rate) can be very high when the study is based on small-size samples (often less than 50 patients) [26]. To improve the reliability of our results, we chose data sets with more than 50 patients (in particular, the largest data set, the TCGA cohort includes a total of 548 patients), used 5 different classifiers during the data mining and carefully validated the results in different data sets. The 5-gene signature consists of CYB561D2, HOXC10, RPL36A, RPS4XP2 and LOC101928747. Among them, CYB561D2 is a member of the cytochrome b561 family, being a hydrophobic, transmembrane heme protein. It is capable of oxidationreduction reaction and is a candidate tumor suppressor gene [27, 28]. HOXC10 belongs to the homeobox family which encodes a highly conserved family of transcription factors that play an important role in morphogenesis, cell differentiation and proliferation. HOXC10 dys-function is found in thyroid cancer [29], breast cancer [30] and cervical squamous cell carcinomas [31]. RPL36A encodes a ribosomal protein that is a component of the 60S subunit of cytoplasmic ribosomes. The protein, which shares sequence similarity with yeast ribosomal protein L44, belongs to the L44E (L36AE) family of ribosomal proteins. RPL36A over-expression is found in hepatocellular carcinoma [32]. For RPS4XP2 and LOC101928747, their function is almost unknown. The biological function of these 5 genes in gliomas might be of great importance for understanding the molecular mechanisms of radioresistance and potential biomarkers in predicting prognosis in gliomas. Taken together, it suggests that both tumor suppressors and oncogenes may affect the prognosis of gliomas. In summary, we have shown that the gene expression profiles of glioma tissues are different between patients that received radiotherapy and patients that didn’t received radiotherapy. Furthermore, we obtained a 20-gene signature associated with radiotherapy in gliomas and a 5-gene signature as an independent predictor of glioma patients’ prognosis. One limitation of this study is that treatment response was not evaluated because this information was not available in most cases. The potential of the 5-gene signature as a biomarker for radioresistance in gliomas deserves validation in the future study.

## MATERIALS AND METHODS

### Data sets of gliomas

A total of 4 independent data sets of gliomas including GSE13041 [33], GSE7696 [34], GSE16011 [35] and the TCGA cohort [36] were downloaded and analyzed. Among them, GSE13041, GSE7696 and GSE16011 were downloaded from the GEO database (Gene Expression Omnibus, http://www.ncbi.nlm.nih.gov/geo/), and the TCGA cohort was downloaded from the TCGA database. GSE13041 has 2 subsets. One subset with 23 patients receiving radiotherapy and 168 patients not receiving radiotherapy was profiled with Affymetrix Human Genome U133A Array [HGU133A] platform. The other one with 17 patients receiving radiotherapy and 10 patients not receiving radiotherapy was profiled with Affymetrix Human Genome U133 Plus 2.0 Array [HG-U133_Plus_2] platform. The clinical outcome information of the 23 patients receiving radiotherapy in the [HG-U133A] subset and the 17 patients receiving radiotherapy in the [HG-U133_Plus_2] subset was available. GSE7696 was profiled with [HG-U133_Plus_2] platform and included 70 radiosensitive patients and 10 radioresistant patients. The clinical outcome information of all the 80 patients was available. GSE16011was profiled with [HGU133_Plus_2] platform and it was used only for the prognosis analysis with a total of 260 patients. The TCGA cohort was profiled with [HG-U133A] platform and included 460 patients receiving radiotherapy and 88 patients not receiving radiotherapy. The clinical outcome information of 459 of the 460 patients receiving radiotherapy was available. The status of radiotherapy is shown in Supplementary Table 2.

### Data processing

After the CEL file of each data set was downloaded, the background was corrected. The raw probe intensities were normalized with the Robust Multichip Average (RMA) [37] method and converted into standardized expression data. Then, we found 13238 common genes among the two platforms and they were used to screen markers of gliomas in subsequent analysis. For genes with more than one probe, the average probe intensity of the same gene was used to calculate its expression value. In order to avoid the systematic error between different platforms, each data set was standardized independently by transforming the expression of each gene to a mean of 0 and SD of 1. The expression profiles were pooled together and then considered them as a single data set [38].

### Identification of a gene signature associated with radiotherapy

GSE13041 was defined as the training set, while GSE7696 and the TCGA cohort were defined as the validation sets. First, 5 different classifiers including Compound Covariate classifier [39], Diagonal Linear Discriminant Analysis (DLDA) classifier [40], Bayesian CCP classifier, Nearest Neighbor classifier (1-Nearest Neighbor & 1-Nearest Neighbor) [41] and Nearest Centroid classifier, were used to re-classify patients receiving radiotherapy (radiation group) and patients not receiving radiotherapy (no radiation group) for exploring specific gene markers that could efficiently separate radiation group from the no radiation group. Among the 5 classifiers, Compound Covariate classifier and DLDA are linear classifiers. During this process, “leave one out cross validation” was used to increase the accuracy and stability of the results. With this method, a total of 20 genes with classification error rate less than 0.16 were identified as genes that were associated with radiotherapy in gliomas in the training set. Then, the accuracy, sensitivity, specificity, positive predictive value (PPV) and negative predictive value (NPV) of the 20-gene signature in separating the radiation group from the no radiation group in different classifiers were calculated. The hierarchical clustering analysis [42] was performed to visually evaluate the expression of the 20-gene signature between these two groups. Hierarchical clustering analysis of gene expression profiles was done based on centered correlation metric and average linkage method. Also, to evaluate the comprehensive ability to separate these two groups, the receiver operating characteristic (ROC) curves were graphed and area under the curve (AUC) was calculated in these two linear classifiers. Next, two validation sets were used to validate the results in the training set. Moreover, the ability of the 20-gene signature in separating these two groups was evaluated by calculating the accuracy in different classifiers, hierarchical clustering analysis and ROC curves.

### Identification of a gene signature associated with prognosis of glioma patients

A total of 4 datasets were divided into the training set (GSE13041) and validation sets (GSE7696, GSE16011, and the TCGA cohort). The training set was used to detect a gene signature associated with prognosis of glioma patients, and the validation sets were used to verify the reliability of this gene signature. In the training set, univariable Cox proportional hazards regression analysis [43] was used. When random permutation test was used and genes with P values less than 0.001 were selected, we obtained a 5-gene signature from the above 20-gene signature. Then, the random survival forests algorithm [44–46] was performed to evaluate the relative importance of each gene to further screen genes associated with the survival of the patients from the 5-gene signature. In this process, number of trees (N tree) was set as 1000, and genes with relative importance more than 0.1 were selected. In fact, the 5 genes were all confirmed to have relative importance more than 0.1. Thus, all the 5 genes were included for subsequent analysis. Then, a risk score model as described previously [46] was constructed using a multivariable Cox regression model based on the 5-gene signature. Risk score of each patient in the training and validation sets was calculated. Patients in the training set and the validation sets were divided into highrisk and low-risk groups using the median risk score as the cut-off. Then the Kaplan-Meier curves were used to further evaluate the difference of overall survival between the two groups, and the hierarchical clustering analysis was performed to visually evaluate the expression of the 5-gene signature between these two groups. Differences in survival time between the low-risk and high-risk groups in each data set were then compared using the two-sided Log Rank (Mantel-Cox) test. Finally, the risk score, together with other clinicopathological parameters were analyzed in univariate and multivariable Cox regression model to verify whether the risk score based on the 5-gene signature is an independent predictor of glioma patients’ prognosis in the training set and the validation sets.

### Gene set enrichment analysis (GSEA)

GSEA was performed using MSigDB C2 CP: Canonical pathways gene set collection. Biological pathways and processes with relative high NES values were considered to be significantly enriched. Enrichment Map was used for visualizing the GSEA results.

### Statistical analysis

The data mining was performed with R software, while other statistical analysis was performed by SPSS (version 17.0). The ROC curves were used to evaluate the ability of the gene signature to separate the radiotherapy group from the no radiotherapy group and AUC of each curve was calculated. The Kaplan-Meier curves were used to evaluate overall survival of the high-risk group and the low-risk group, along with the two-sided Log Rank (Mantel-Cox) test to determine if the difference between the two groups was significant. Other statistical methods included the Cox proportional hazard models, univariate and multivariable Cox regression model. In this study, all statistical tests were two-tailed and differences were considered statistically significant if *P*-values<0.05.

## SUPPLEMENTARY MATERIALS TABLES





## Abbreviations

(AML): acute myeloid leukemia
(AUC): area under the curve
(DEGs): differentially expressed genes
(DLDA): Diagonal Linear Discriminant Analysis
(FDR): False Discovery Rate
(GEO): Gene Expression Omnibus
(GBM): glioblastoma
(IR): ionizing radiation
(NPV): negative predictive value
(OS): overall survival
(PPV): positive predictive value
(RMA): Robust Multichip Average
(ROC): receiver operating characteristic
(WHO): World Health Organization
