# Supplementary material for: Identification of a gene signature associated with radiotherapy and prognosis in gliomas
Source: Oncotarget. 2017 Oct 6;8(51):88974–87. doi: 10.18632/oncotarget.21634 (PMC5687662; doi:10.18632/oncotarget.21634)
Supplement: Supplementary file 1 [file oncotarget-08-88974-s001.pdf]

Identification of a gene signature associated with radiotherapy and prognosis in gliomas

SUPPLEMENTARY MATERIALS

Supplementary Table 1: The gene list of the 20-gene signature associated with radiotherapy in gliomas

Genes
ANAPC1
BTBD7
CA11
CYB561D2
DRD5
FKBP6
HOXC10
LAMB4
LOC101928747
PADI1
PAX3
PF4
PYGM
QPCTL
RPL36A
RPS4XP2
SLC18A1
TP53TG3
USB1
ZNF280A

Supplementary Table 2: The status of radiotherapy in datasets

See Supplementary File 1
